# Terahertz Resonant Emission by Optically Excited Infrared‐Active Shear Phonons in KY(MoO_4_)_2_

**DOI:** 10.1002/advs.202407028

**Published:** 2024-11-13

**Authors:** Dmytro Kamenskyi, Kirill Vasin, Lilian Prodan, Khrystyna Kutko, Volodymyr Khrustalyov, Sergey G. Pavlov, Heinz‐Wilhelm Hübers

**Affiliations:** ^1^ Institute of Optical Sensor Systems German Aerospace Center (DLR) Rutherfordstr. 2 12489 Berlin Germany; ^2^ Department of Physics Humboldt‐Universität zu Berlin Newtonstr. 15 12489 Berlin Germany; ^3^ Experimental Physics V Center for Electronic Correlations and Magnetism Institute of Physics University of Augsburg Universitätsstr. 1 86159 Augsburg Germany; ^4^ B. Verkin Institute for Low Temperature Physics and Engineering of the National Academy of Sciences of Ukraine Nauky Avenue 47 Kharkiv 61103 Ukraine

**Keywords:** coherent phonons, infrared‐active phonons, molybdates, narrowband emission, shear lattice vibrations, terahertz radiation

## Abstract

The generation of monochromatic electromagnetic radiation in the terahertz (THz) frequency range has remained a challenging task for many decades. Here, the emission of monochromatic sub‐THz radiation by optical phonons in the dielectric material KY(MoO_4_)_2_ is demonstrated. The layered crystal structure of KY(MoO_4_)_2_ causes infrared‐active shear lattice vibrations to have energies below 3.7 meV, corresponding to frequencies lower than 900 GHz where solid‐state‐based monochromatic radiation sources are rare. Directly excited by a 5 ps long broadband THz pulse, infrared‐active optical vibrations in KY(MoO_4_)_2_ re‐emit narrowband sub‐THz radiation as a time‐varying dipole for tens of picoseconds, which is exceptionally long for oscillators with frequencies below 1 THz. Such a long coherent emission allows for the detection of more than 50 periods of radiation with frequencies of 568 and 860 GHz. The remarkably long decay time together with the chemical stability of the employed material suggests a variety of possible applications in THz technology.

## Graphical abstract

Researchers demonstrate that KY(MoO_4_)_2_ crystals emit narrowband sub‐terahertz radiation when excited by a broadband terahertz pulse. The material's unique optical phonons re‐emit radiation at frequencies of 568 and 860 GHz, lasting over 100 picoseconds. This long‐lasting, monochromatic emission opens new possibilities for applications in terahertz technology.

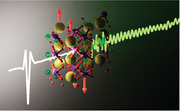

## 1 Introduction

Over the last decades, significant advancements in Time‐Domain Terahertz Spectroscopy (THz‐TDS) have boosted interest in terahertz (THz) research and its applications.^[^ ^1^ ^]^ THz‐TDS is based on electromagnetic transients optoelectronically generated by ultrashort, usually femtosecond, laser pulses. The single‐cycle bursts of electromagnetic radiation typically have durations of about 1 ps with spectral density enhanced between 100 GHz and 5 THz.^[^ ^2^ ^]^

It is well known that a time‐varying electric dipole moment emits electromagnetic waves at the oscillation frequency of the dipole.^[^ ^2^ ^]^ Coherent excitation of the lattice vibrations can therefore lead to the emission on the frequency of these vibrations. It has been shown that propagation of light pulses in solids could be accompanied by intense THz lattice vibrations with a high spatial and temporal coherency, which in turn leads to a variety of possible applications.^[^ ^3^, ^4^, ^5^ ^]^ For the first time, the photo‐Dember effect of electromagnetic wave re‐emission by coherent phonons in the THz range of frequencies was reported in tellurium single crystals.^[^ ^6^ ^]^ Recently, THz emission was also reported in hybrid perovskites,^[^ ^7^ ^]^ charge‐density wave system K_0.3_MoO_3_,^[^ ^8^ ^]^ and the topological material TaAs, where it offers opportunities for THz emission with polarization control.^[^ ^9^ ^]^ A recent overview of THz re‐emission by optically pumped solids is given elsewhere.^[^ ^10^ ^]^ Generally, the photo‐Dember THz re‐emission is broadband with typical spectrum widths above 100 GHz.

Despite this progress, the generation of monochromatic THz radiation remains a challenging task. However, for applications such as THz imaging,^[^ ^11^ ^]^ THz‐driven particle acceleration,^[^ ^12^ ^]^ or ultrafast phase changes,^[^ ^13^ ^]^ narrowband THz pulses are crucial. Lithium niobate^[^ ^14^ ^]^ as well as other materials like semiconductors^[^ ^15^, ^16^ ^]^ and organic crystals^[^ ^17^ ^]^ have been reported as perspective materials for the generation of high‐energy THz pulses with durations of tens of picoseconds and typical bandwidths of about 1%.

**Figure 1:**
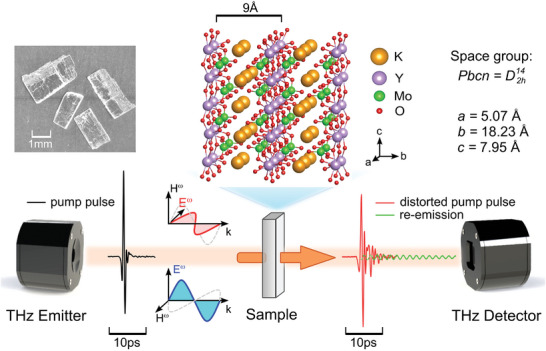
Top: Photograph and crystallographic structure of KY(MoO_4_)_2_ single crystal. Bottom: Schematic representation of the THz‐TDS setup. The black line shows the trace of the THz waveform from the THz emitter. Red and green curves show the components of the beam entering the detector. The cryogenic part of the setup is omitted for clarity. *E* ^ω^ stands for the electric field magnitude of the electromagnetic wave.

Here we present observations of long‐lasting (above 100 ps), a narrowband (full width at half maximum Δν < 4 GHz or 0.5%) re‐emission of THz electromagnetic pulses by KY(MoO_4_)_2_ single crystals pumped by a broadband THz excitation pulse with spectral range 0.1–2.5 THz. KY(MoO_4_)_2_ belongs to a series of molybdate compounds with the general chemical formula *MR*(MoO_4_)_2_, where *M* ^+^ is an alkali metal ion and *R* ^3 +^ is a rare‐earth or Y^3 +^ ion. These are optically transparent dielectric compounds with a high permittivity (ϵ > 15) and orthorhombic structure *Pbcn* (*D* 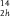), which is formed by [*R*(MoO_4_)_2_]^−^ layers in the *ac* plane coupled via K^+^ ions along the *b* direction (see crystallographic structure in **Figure** **1**).^[^ ^18^ ^]^

In recent years, rare‐earth‐based molybdates have attracted attention due to a giant rotational magnetocaloric^[^ ^19^ ^]^ and massive magnetostriction^[^ ^20^ ^]^ effects induced by large anisotropy of the *R* ^3 +^ ion. Here, we exploit the large anisotropy of the crystal lattice of KY(MoO_4_)_2_ for the emission of THz radiation. In our experiments, we used plate‐shaped single crystals (Figure 1 left) with the crystallographic *b* axis perpendicular to the plates. Due to weak bonding between [*R*(MoO_4_)_2_]^−^ layers, single crystals cleave along the *ac* plane.

## 2 Experimental Section

### 2.1 Sample Preparation

KY(MoO_4_)_2_ single crystals were grown using the top‐seeded solution growth method^[^ ^21^ ^]^ at the Institute for Low Temperature Physics (Kharkiv, Ukraine). The detailed description of the synthesis is given in Ref. [21]. The single crystals were cleaved along the *ac* plane to obtain plate‐shaped specimens with thicknesses ranging from 65 to 150 µm. The compositional analysis of the crystals was done using scanning electron microscopy in combination with energy‐dispersive X‐ray spectroscopy and elemental mapping.

### 2.2 THz Time‐Domain Spectroscopy

THz‐TDS measurements were conducted using a TeraFlash time‐domain terahertz platform (TOPTICA Photonics AG). The radiation propagated along the *b*‐axis of the crystal, and measurements were performed for polarizations *E* ^ω^∥*a* and *E* ^ω^∥*c*. The data were analyzed using the REFFIT script to extract the complex dielectric function Ref. [24].

Figure 1 depicts a sketch of the experimental setup, the image of samples, and the crystallographic structure of KY(MoO_4_)_2_ crystal. The specimen was mounted to a brass holder with 4 mm aperture inside the Spectromag cryostat (Oxford Instruments) (which is omitted in the Figure 1 for clarity) with an insert which allows temperature variation between 3 and 300 K. The black curve shown in front of the sample represents a temporal profile of electric field during the THz pulse incident on the sample (pump signal), which is equal to whose measured when the radiation passed the empty aperture (reference signal). This pump pulse lasts for about 5 ps and has a spectrum between 0.1 and 2.5 THz. Curves behind the sample show the THz profile measured when the pulse passed the sample. After it passes the sample, the pulse contains contributions of the distorted initial pump pulse (red curve) and the radiation re‐emitted by the crystal (green curve), further the microscopic mechanism of this re‐emission is discussed. The square of the ratio of the Fourier transforms of the measured transients (passed the sample over the reference) returns the sample's transmittance spectrum (transmitted energy). Taking this ratio as a transmittance, it is assumed that the sample does not affect significantly the efficiency of the light collection by the detector.

## 3 Results and Discussion

### 3.1 Transmittance and Absorption Spectra

**Figure 2:**
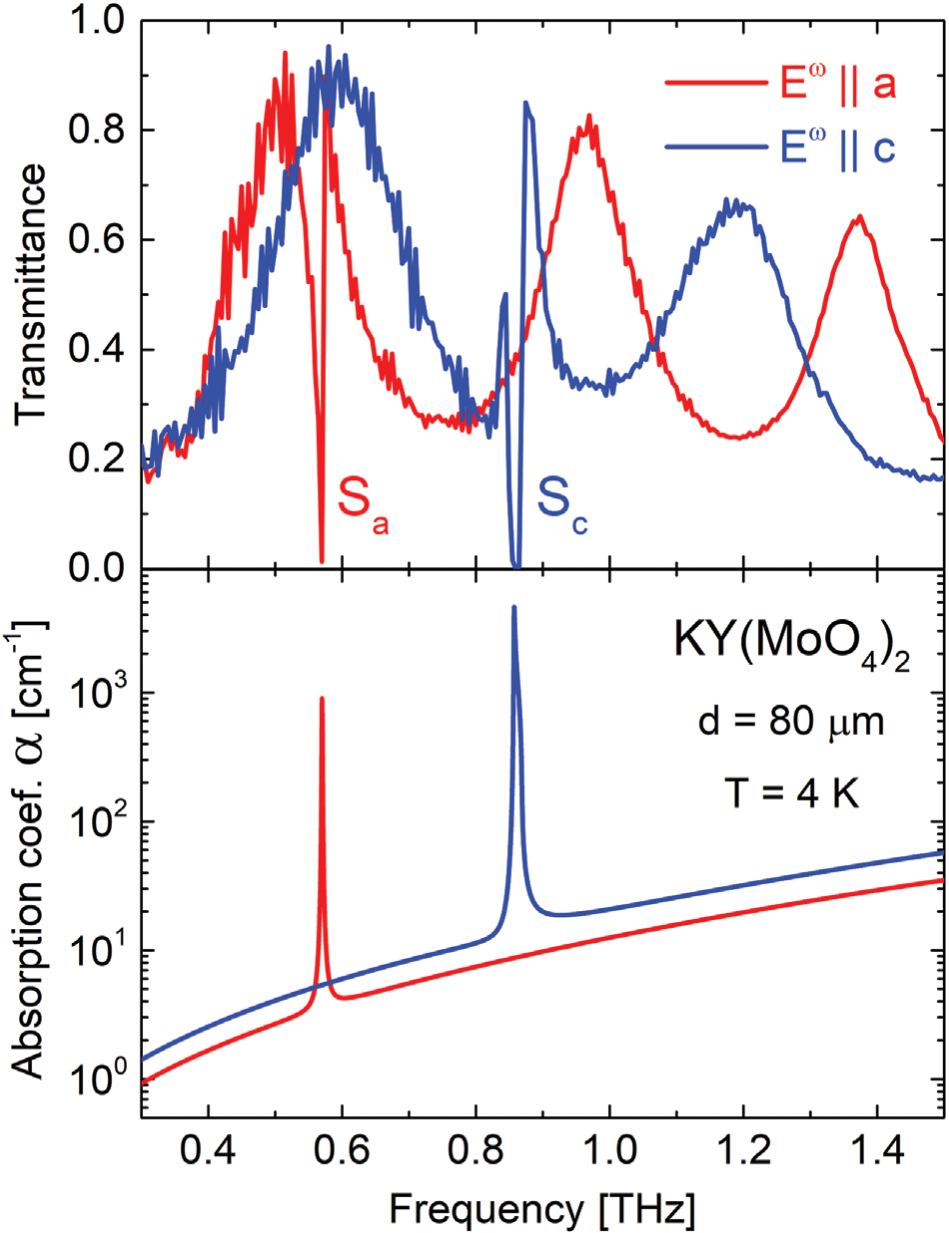
Top: Transmittance spectra of an 80 µm thick KY(MoO_4_)_2_ sample for *E* ^ω^∥*a* (red) and *E* ^ω^∥*c* (blue) polarizations. Multiple reflections within the plane‐parallel sample result in Fabry–Pérot type modulation of spectra. Bottom: Frequency dependence of the absorption coefficient α(ω) obtained using the REFFIT script.^[^ ^24^ ^]^

**Figure** **2** (top) displays the transmittance spectra of an 80 µm thick KY(MoO_4_)_2_ single crystal at frequencies between 0.3 and 1.5 THz measured at 4 K. Red and blue curves correspond to the THz wave polarized along the *a* and *c* crystallographic axes, respectively (*E* ^ω^∥*a* and *E* ^ω^∥*c*, where *E* ^ω^ is the electric field magnitude of the electromagnetic wave). Sharp peaks *S*_*a*_ and *S*_*c*_ at 568 and 860 GHz (phonon energy equivalents are 2.35 and 3.56 meV), respectively, are due to dipole‐active shear lattice vibrations, when [Y(MoO_4_)_2_]^−^ and K^+^ layers move as a whole in the *ac* plane, as has been shown earlier.^[^ ^18^ ^]^ A large mass of [Y(MoO_4_)_2_]^−^ layers together with weak bonding between them causes the frequencies of these vibrations to be below 1 THz, while typical optical phonons in solids have frequencies above 3 THz.^[^ ^22^, ^23^ ^]^

Another important peculiarity of *MR*(MoO_4_)_2_ compounds is that the frequencies of the shear vibrations are considerably lower than those of other phonons in these compounds. The next infrared‐active vibrations are at 2.58 THz for *E* ^ω^∥*a* and 3.36 THz for *E* ^ω^∥*c*.^[^ ^18^ ^]^ Such a big gap between phonon energies significantly reduces the dissipation rate as comparable for semiconductors.^[^ ^23^ ^]^ Remarkably, the *S*_*c*_ absorption is about ten times stronger than *S*_*a*_, making the sample opaque for *E* ^ω^∥*c* radiation with frequencies in the vicinity of the *S*_*c*_ peak (Figure 2), and causing a peculiarity in the re‐emission spectrum of the *S*_*c*_ phonon, which we discuss further.

Multiple reflections of the radiation within the plane‐parallel sample result in interference fringes of the transmission spectra (Figure 2 top). The periodicity of the fringes is determined by the thickness of the crystal, *d*, and the sample's refractive index, *n*. We used REFFIT script^[^ ^24^ ^]^ to determine the complex dielectric function of the material, $\hat{\varepsilon }\left(\omega \right)=\varepsilon_{1}-i\varepsilon_{2}$, and, consequently, the frequency dependence of the refractive index, *n*, the optical extinction coefficient, κ, and the absorption coefficient $\alpha \left(\omega \right)=2\kappa \omega /c_{0}=2\omega /c_{0}\sqrt{1/2\cdot {\left(\varepsilon_{1}^{2}+\varepsilon_{2}^{2}\right)}^{1/2}-\varepsilon_{1}/2}$ (Figure 2 bottom) where *c*_0_ is the speed of light in vacuum. According to the Beer–Lambert law, *I*(*d*) = *I*_0_ *e* ^−α · *d*^ (*d* is the sample thickness), 1/α corresponds to the sample thickness when the light intensity, *I*, decays in *e* times.

The extremely narrow line width of the peaks (for *E* ^ω^∥*a* it is below the reliable resolution limit of 5 GHz) reflects the exceptionally long lifetime of such a dipole. To the best of our knowledge, these are the sharpest phonon peaks ever observed below 1 THz. The lifetime could be estimated as τ = (πΔν)^−1^ ≈ 100 ps^[^ ^25^, ^26^ ^]^ (where Δν is the full width at half maximum of *S*_*a*_ mode in the α(ω) spectrum, Figure 2 bottom). Thus, τ of *S*_*a*_ phonon is an order of magnitude exceeds the usual phonons lifetime (typically 1 − 10 ps) as well as the duration of the excitation THz pulse in our setup (∼5 ps). For the *S*_*c*_ phonon such estimation is not possible because of the strong absorption in a vicinity of *S*_*c*_ and the transmitted signal between 857 and 863 GHz is below the noise level of the detector and the peak is distorted (later in the text we call such distortion of the peak *saturation*).

### 3.2 Time‐Domain Spectroscopy and Re‐Emission

**Figure 3:**
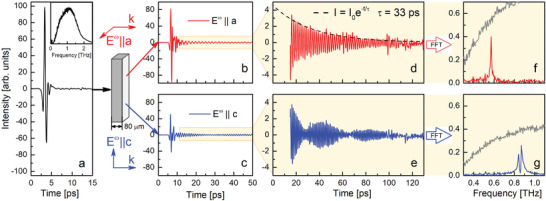
a) Electric field waveform of the THz pulse before it enters the sample. The inset shows the FFT spectrum of this waveform. b,c) Waveforms of the THz pulse after passing through the sample for polarizations along *a* (red) and *c* (blue) axes (Measurements are done at 4 K). d,e) Zoomed waveforms for the *a* (red) and *c* (blue) polarizations 10 ps after the start of the pulse. f,g) FFT of the waveforms is shown in (d) and (e), respectively. Grey lines are FFT of the signal before the sample. Curves.

**Figure** **3**a shows the electric field waveform of the broadband THz pump/reference pulse (its Fourier Transform (FFT) spectrum is displayed in the inset). The signal decays within 4–5 ps, and the shape of this pulse is identical to the pulse before it passes the sample (losses in optical elements are assumed to be the same for the beams passing the cryostat with and without sample). The waveforms that passed through the 80 µm thick sample (Figures 3b,c for *E* ^ω^∥*a* and *E* ^ω^∥*c*, respectively) exhibit oscillations on phonon frequencies for tens of picoseconds. Note, the transmitted signal is also delayed by 1 ps due to a large refractive index of KY(MoO_4_)_2_, *nd*/*c*_0_ ∼ 1 ps (*n* ≈ 4 below 1 THz).

For *E* ^ω^∥*a*, the waveform shows oscillations with a decay an order of magnitude longer than the initial pulse (highlighted by the yellow background in Figure 3b and zoomed in the Figure 3d). The dashed line in Figure 3d represents the exponential decay of radiation intensity fitted by exp (− *t*/τ_*e*_), where τ_*e*_ = 33 ps is the decay time. Such a long decay and significant intensity of the signal allow the observation of tens of optical periods of a monochromatic electromagnetic wave. Figure 3f,g shows FFT spectra of the waveform depicted in 3d,e. Grey lines in 3d,e display the spectrum of the radiation before the sample. A single peak in the curve on Figure 3f confirms that after the initial pulse passed, the electromagnetic radiation with *E* ^ω^∥*a* becomes quasi‐monochromatic light with the frequency *f* = 568 GHz, which is identical to the absorption frequency of the *S*_*a*_ phonon. A comparison of the radiation intensities on the *S*_*a*_ frequency before and after the sample reveals that approximately 80% of the initial radiation is re‐emitted within the subsequent 100 ps. Thus, long extended emission in temporal profile in Figure 3b,d is a manifestation of the electromagnetic wave re‐emission by phonons in KY(MoO_4_)_2_.

In contrast, the waveform for *E* ^ω^∥*c* (Figure 3c) after the main peak exhibits a beating of two frequencies (Figure 3e). In the FFT, this leads to a sharp dip in the re‐emission spectrum at the frequency of the *S*_*c*_ phonon (Figure 3g). Subsequently, we demonstrate that this is the consequence of the saturation observed in the transmittance spectra (Figure 2 top, blue curve). The strong intensity of *S*_*c*_ phonon prohibits the propagation of the resonance frequency within the material while the lattice vibrations at frequencies in close proximity to the resonance efficiently re‐emit the electromagnetic radiation.

### 3.3 Temperature and Thickness Dependence

**Figure 4:**
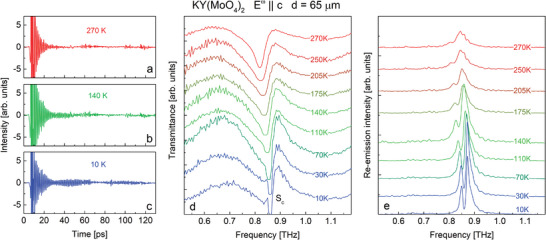
Temperature evolution of the waveforms (a, b, c), transmittance (d) and re‐emission (e) spectra for the 65 µm thick KY(MoO_4_)_2_ sample at *E* ^ω^∥*c*. Here, we call the FFT as “re‐emission” after a 10 ps delay from the pump pulse front.

To confirm that the two‐peak structure of the re‐emission signal for *E* ^ω^∥*c* is due to the strong intensity of the *S*_*c*_ phonon, we conducted a study on the temperature transformation of the spectrum. **Figure** **4** displays the evolution of waveforms (left), transmittance (middle), and re‐emission (right) spectra of KY(MoO_4_)_2_ for *E* ^ω^∥*c* upon cooling. The re‐emission spectra were obtained in the same manner as in Figure 3f,g. As the temperature decreases, the phonon peak *S*_*c*_ narrows and grows in amplitude, which leads to the development of the re‐emission peak, which splits simultaneously with saturation in the transmittance peaks. As one can see in Figure 4, the re‐emission spectrum at room temperature (270 K) exhibits a quasi‐monochromatic Lorentzian shape. Upon cooling, the frequencies where the transmittance hits zero are missing in the re‐emission spectrum, which leads to the formation of a sharp dip in the emission peak. We note, that remarkable re‐emission at room temperature significantly enlarges the application perspectives.

**Figure 5:**
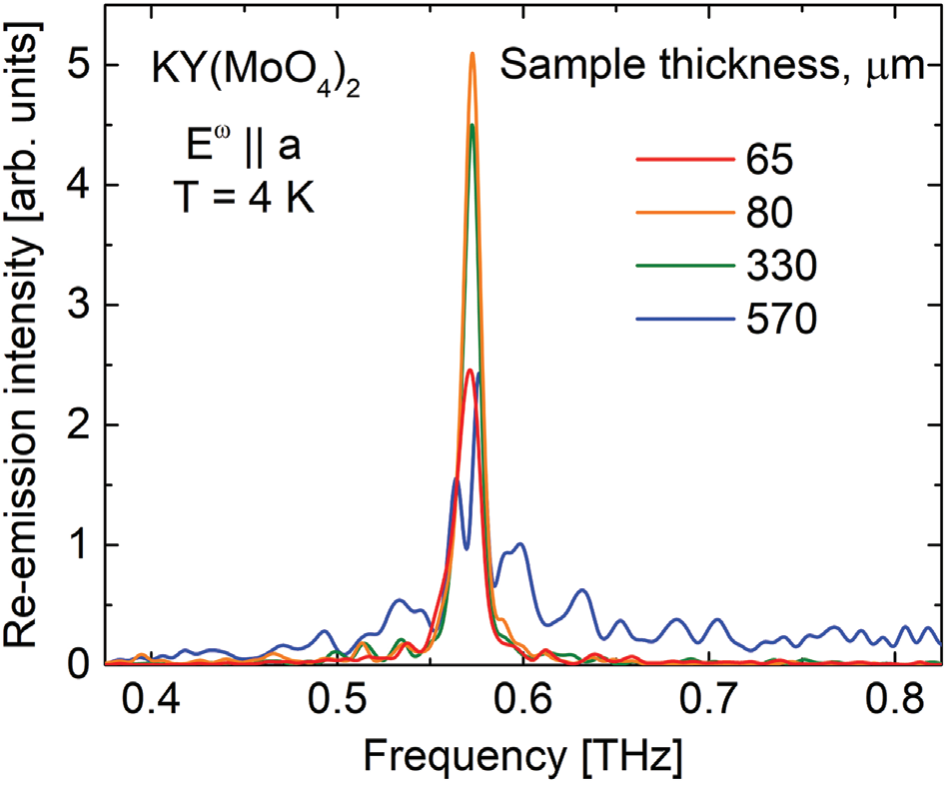
Thickness evolution of the re‐emission spectra of KY(MoO_4_)_2_ at 4 K and *E* ^ω^∥*a*.

Similar peak splitting is observed in the thickness dependence of the re‐emission spectrum (**Figure** **5** for *E* ^ω^∥*a*). One can see that the maximal re‐emission intensity of the 65 µm thick sample is significantly smaller than that of the 80 µm one. These thicknesses are not far from the value of 1/α_*a*_ ≈ 10 µm at *S*_*a*_ frequency When the sample thickness increases further, the intensity of the re‐emission decreases because the radiation spends more time inside the material, and the energy dissipates into other optical and acoustic phonons.

When *d* ≫ 1/α by an order of magnitude the re‐emission intensity on the central frequency decays, while on frequencies near the resonance, the re‐emission remains efficient as it has been described above for the *E* ^ω^∥*c* polarization. This leads to the beating in the time‐domain spectra (Figure 4e) and to the spectral dip in the re‐emission peak (Figure 4g).

Beating shapes of TDS transients propagating through the media with strong, spectrally narrow absorption peaks have attracted the attention of many theoretical groups in the past. Different phenomena, such as so‐called Sommerfield–Brillouin forerunners,^[^ ^27^ ^]^ dynamical beats, and optical precursors generated by the incident step‐modulated pulses, were utilized to explain similar experimental observations. We point to a comprehensive work by Bruno and Bernard^[^ ^28^ ^]^ focused on the response function of the dielectric medium with a single absorption line. It outlines the conditions for observing dynamical beats: 1 ≪ α*d* ≪ ν_0_/Δν, where ν_0_ represents the central frequency of the absorption line, and *d* stands for the thickness of the slab. In our case, for an 80 µm sample at 4 K, these results are 1 ≪ 7 ≪ 475 for the *S*_*a*_ phonon and 1 ≪ 37 ≪ 329 for the *S*_*c*_ phonon. Thus, for both polarizations, the condition is met. However, a detailed discussion of the microscopic mechanism of the beating formation would require significantly higher spectral resolution, which is not feasible for the current TDS spectroscopy and goes beyond the scope of this study, where we focus on the optimal conditions for the monochromatic THz re‐emission.

### 3.4 Discussion

In KY(MoO_4_)_2_, the radiation energy absorbed by the infrared‐active optical *S*_*a*_ phonon during the initial broadband THz excitation pulse (∼5 ps) is not dissipated into the lattice via phonon–phonon interactions due to limited coupling of these shear vibrations to other phonons. Instead, most of this energy is re‐emitted via the mechanism of a time‐varying dipole by the coherent dipole‐active lattice vibrations as monochromatic THz radiation.^[^ ^2^ ^]^

Such coherent photon re‐emission has been reported previously in different classes of materials ranging from elemental semiconductor tellurium^[^ ^6^ ^]^ to hybrid perovskites^[^ ^7^ ^]^ and the topological material TaAs,^[^ ^9^ ^]^ but usually at frequencies above 1 THz and in rather broad spectra. In our experiments, however, exceptionally low phonon energies and long lifetimes allow the detection of extremely narrowband re‐emissions with central frequencies of 568 GHz and 860 GHz for *E* ^ω^∥*a* and *c* crystallographic axes, respectively, with a decay time of 33 ps. For *E* ^ω^∥*a*, the emission lasts for more than 100 ps or more than 50 optical cycles. These unique characteristics, together with the chemical durability, make KY(MoO_4_)_2_ very attractive for various THz applications, ranging from conventional magnetic resonance spectroscopy^[^ ^29^ ^]^ to narrowband THz pulse generation for novel THz‐driven electron acceleration systems.^[^ ^30^ ^]^

Other materials from the family of *MR*(MoO_4_)_2_ exhibit similar phonon spectra below 1 THz. All these compounds have *S*_*a*_ and *S*_*c*_ phonons similar to those discussed above, with small frequency variations due to different masses of *R* ^3 +^ and *M* ^+^ ions.^[^ ^18^ ^]^ Particularly, similar time traces were observed in KTm(MoO_4_)_2_ and KEr(MoO_4_)_2_ compounds. This creates a versatile ground for the development of sub‐THz radiation sources. An additional significant advantage is the chemical stability of these materials; they may be kept under ambient laboratory conditions for decades without changing composition, crystallographic structure, or other physical properties. Our experiments have revealed no difference between samples obtained in 2024 and in 1980th. *MR*(MoO_4_)_2_ compounds are resistant to standard solvents such as water, acetone, and alcohol, which significantly simplify a working protocol.

## 4 Conclusion

We have demonstrated that KY(MoO_4_)_2_ single crystals exhibit long‐lasting, narrowband re‐emission of THz electromagnetic pulses when pumped by a broadband THz excitation pulse. The exceptionally low energies and long lifetimes of the infrared‐active shear phonons enable the observation of re‐emission at frequencies of 568 and 860 GHz with decay times exceeding 33 ps. These findings open up new possibilities for generating monochromatic THz radiation using chemically stable dielectric materials.

## Conflict of Interest

The authors declare no conflict of interest.

## Data Availability

The data that support the findings of this study are available from the corresponding author upon reasonable request.
